# Supplementary material for: Rapid turnover of life-cycle-related genes in the brown algae
Source: Genome Biol. 2019 Feb 14;20:35. doi: 10.1186/s13059-019-1630-6 (PMC6374913; doi:10.1186/s13059-019-1630-6)
Supplement: Supplementary file 3 — Protein alignments, corresponding Gblocks html file and CDS sequences for the lineage conserved gametophyte-biased genes. (ZIP 2109 kb) [file 13059_2019_1630_MOESM3_ESM.zip › Con_GA_alignments/Ec-01_006190.aa_ali.fasta-gb.htm]

Ec-01\_006190.aa.fasta.trans.aa\_ali.fasta


## Gblocks 0.91b Results

Processed file: **Ec-01\_006190.aa.fasta.trans.aa\_ali.fasta**  
Number of sequences: **2**  
Alignment assumed to be: **Protein**  
New number of positions: **447** (selected positions are underlined in blue)

```
                         10        20        30        40        50        60
                 =========+=========+=========+=========+=========+=========+
Ec-01_006190     MAGVGSDATELKLHSCIGFTGKVLGGMKLTPCGGYMVYPLGSILVVRSMTGKNKQAFLEG
MSTRG.29191.1    -----------------------LGGMKSTPCGGYMVYPLGSILVVRSMTGKNKQAFLEG
                                        #####################################


                         70        80        90       100       110       120
                 =========+=========+=========+=========+=========+=========+
Ec-01_006190     HTSEISCVAMSSDGTRIVSGQANHMGVKADLCVWDLIQAQANCDAEKPSGAGVLLHRLKQ
MSTRG.29191.1    HTSEISCVAMSSDGDRIVSGQANHMGVKADLCVWDLIQAQANCDADRPNGAGVLLHRLKQ
                 ############################################################


                        130       140       150       160       170       180
                 =========+=========+=========+=========+=========+=========+
Ec-01_006190     HLGHVRAVDFSFDGKYFASLGGQDDNSVVIWEAKTGRAVCGSPAAQDSALSVKWLHRRND
MSTRG.29191.1    HLGHVRAVDFSFDGKYFASLGGQDDNSVVIWETDTGRAVCGSPAAQDSAFSVKWLHRRND
                 ############################################################


                        190       200       210       220       230       240
                 =========+=========+=========+=========+=========+=========+
Ec-01_006190     RFVTAGNFHLRVWQVDVSIPKVHAVDAQMGGMRRAIECISITEDDEHAYCGTRTGDVLKF
MSTRG.29191.1    RFVTAGNFHLRVWQVDVSIPKVHAVDAQMGGMRRTIECISITEDDKHAYCGTRTGDVLKF
                 ############################################################


                        250       260       270       280       290       300
                 =========+=========+=========+=========+=========+=========+
Ec-01_006190     KIDRDDIRYFNDPDTIRPTLAGYSLEKFGKGVRSVRCVLNPETGNTNTLVGGGDGTVSFI
MSTRG.29191.1    TIDRDDIRYFNDPDTIRPTLAGYSLDKFGKGVRSVCCVLNPETGNTNTLVGGGDGTVSFI
                 ############################################################


                        310       320       330       340       350       360
                 =========+=========+=========+=========+=========+=========+
Ec-01_006190     NQSLNKVAGKHCELMGAVTSIAVCPGNKGFFVGTALSNRYWVTMDMETELRGTCHFARVN
MSTRG.29191.1    NQSLNKVVGKRCELMGAVTSIAMCPGNKGFFVGTSLSNRYWVTMDMETELRGTCHFARVN
                 ############################################################


                        370       380       390       400       410       420
                 =========+=========+=========+=========+=========+=========+
Ec-01_006190     DIIFPAGCSDLFITCSVADIRVWNATLRQELLRIQVPNLECFCVGITRSGGTIVSGWSDG
MSTRG.29191.1    DIIFPADCPDLFITCSVADIRVWNAALRQELLRIQVPNLECFCVGITRSGGTIVSGWSDG
                 ############################################################


                        430       440       450       460       470       480
                 =========+=========+=========+=========+=========+=========+
Ec-01_006190     KIRAFYPESGKLKFVISDAHADGATALALAQDDDTRPPWRLISGGGDGRVRVWSVTSSHQ
MSTRG.29191.1    KIRAFYPESGKLKFVISDAHAEGTTALALAHDDDTRPPWRIISGGGDGRVX---------
                 ##################################################          


                        490       500       510       520       530       540
                 =========+=========+=========+=========+=========+=========+
Ec-01_006190     AMTTSWKEHRGPVTCIQVSRENGQCISGSADGSCIVWDLNRGVRVLALFEPNVFKGVRYH
MSTRG.29191.1    ------------------------------------------------------------
                                                                             


                        550       560       570       580       590       600
                 =========+=========+=========+=========+=========+=========+
Ec-01_006190     PDESQYITCGSNHKITYWDAYDGTAIRVIEGGDEEMNTLDVEPSGEAFVSGGDDGLVKVW
MSTRG.29191.1    ------------------------------------------------------------
                                                                             


                        610       620       630       640       650       660
                 =========+=========+=========+=========+=========+=========+
Ec-01_006190     HYDDGLTMSVGTGHSGSVAAVRISPDRKIIVSAGSEGGIFIWGMPPPSCDAHAGGSPGRS
MSTRG.29191.1    ------------------------------------------------------------
                                                                             


                        670       680       690
                 =========+=========+=========+====
Ec-01_006190     GATTNGCNLEAVAAGGNLNIDKMADDVSRVSMSR
MSTRG.29191.1    ----------------------------------
```

```
Parameters used
Minimum Number Of Sequences For A Conserved Position: 2
Minimum Number Of Sequences For A Flanking Position: 2
Maximum Number Of Contiguous Nonconserved Positions: 8
Minimum Length Of A Block: 10
Allowed Gap Positions: None
Use Similarity Matrices: Yes
```

```
Flank positions of the 1 selected block(s)
Flanks: [24  470]  

New number of positions in Ec-01_006190.aa.fasta.trans.aa_ali.fasta-gb:  447  (64% of the original 694 positions)
```
